# A paradigm shift in Diastasis Recti surgery: The Bikini-line robotic approach

**DOI:** 10.1590/0100-6991e-20243846-en

**Published:** 2025-11-24

**Authors:** ANDRE LUIZ GIOIA MORRELL, ALLAN GIOIA MORRELL, ALEXANDER CHARLES MORRELL, ALEXANDER CHARLES MORRELL

**Affiliations:** 1 - Instituto Morrell, Cirurgia do Aparelho Digestivo Minimamente Invasiva e Robótica - São Paulo - SP - Brasil; 2 - Hospital Vila Nova Star, Cirurgia Geral e do Aparelho Digestivo Minimamente Invasiva e Robótica - São Paulo - SP - Brasil; 3 - Sociedade Beneficente Israelita Brasileira Albert Einstein, Cirurgia Geral e do Aparelho Digestivo Minimamente Invasiva e Robótica - São Paulo - SP - Brasil

**Keywords:** Diastasis, Muscle, Hernia, Minimally Invasive Surgical Procedures, Robotic Surgical Procedures, Diástase Muscular, Hérnia, Procedimentos Cirúrgicos Robóticos, Procedimentos Cirúrgicos Minimamente Invasivos

## Abstract

**Introduction:**

diastasis recti surgery has been known worldwide for open surgical techniques involving significant tissue manipulation, skin flap and larger incisions. Traditional methods typically required extended recovery times and posed higher risks of complications and scarring issues. The advent of robotic-assisted surgery has revolutionized the treatment paradigm for abdominal wall defects and its remarkable outcomes encouraged expanding its applications towards diastasis recti pathologies. Better visualization and more ergonomic instruments foster a minimal scarring procedure, allowing surgeons to improve aesthetic and recovery outcomes following diastasis recti correction in a posterior approach. This article describes a robotic surgical technique and results to an unprecedented approach, putting its form of treatment into another perspective.

**Technical Report:**

a step-by-step guided technique of this novel technique is described using detailed port placement and figures to assure optimal aesthetic and functional outcomes whenever acting in minimally invasive diastasis recti repair with the da Vinci platform.

**Conclusion:**

The described technique reveals a hidden minimal incisions procedure avoiding skin flaps, scarring issues, and minimizing wound morbidity. Through a step-by-step guide, this report establishes an unprecedent technique description transforming the diastasis recti surgery scenario and its aesthetic outcomes with a safe minimally invasive surgery.

## RESUMO

**Introdução:**

a cirurgia de diástase dos retos abdominais é conhecida mundialmente por técnicas cirúrgicas abertas que envolvem manipulação significativa de tecidos, retalhos de pele e incisões maiores. Os métodos tradicionais normalmente exigem tempos de recuperação prolongados e apresentam maiores riscos de complicações e cicatrização. O advento da cirurgia robótica revolucionou o paradigma de tratamento para defeitos da parede abdominal e seus resultados notáveis encorajaram a expansão de suas aplicações para patologias de diástase dos retos abdominais. Melhor visualização e instrumentos mais ergonômicos promovem um procedimento minimamente invasivo, permitindo que os cirurgiões melhorem os resultados estéticos e de recuperação após a correção da diástase abdominal com uma abordagem posterior. Este artigo descreve uma técnica cirúrgica robótica colocando em outra perspectiva a sua forma de tratamento.

**Nota Técnica:**

uma padronização guiada passo a passo desta técnica é descrita usando posicionamento detalhado de portais e figuras para garantir resultados estéticos e funcionais ideais quando atuando no reparo minimamente invasivo da diástase dos retos abdominais com a plataforma robótica da Vinci.

**Conclusão:**

A técnica descrita revela uma cirurgia de incisões mínimas, minuciosamente ocultas, evitando retalhos de pele, morbidade de cicatrizes e fornece uma restauração fisiológica da parede abdominal. Através de uma padronização passo a passo, este artigo descreve uma técnica para correção de diástase abdominal permitindo resultados funcionais e estéticos satisfatórios com uma cirurgia robótica minimamente invasiva segura.

## INTRODUCTION

Diastasis rectus abdominis is a prevalent condition characterized by the separation of the rectus abdominis muscles, usually resulting from factors such as pregnancy, significant weight changes, or advanced age. Surgical correction of diastasis abdominis has traditionally been based on open abdominoplasty techniques performed through an extensive inferior incision, with the aim of removing excess skin and adipose tissue from the abdominal area, while fastening the underlying musculature^1^. Although the procedure is effective, its drawbacks include extended recovery time, pain, and a risk of complications that may impact patient outcomes and aesthetic satisfaction^2^.

One of the frequently reported complications is the development of seromas, which are collections located at the site of flap detachment, prolonging recovery, which may even require subsequent interventions for drainage^3^. In addition to seromas, patients may sustain complications in their wounds, which usually involve some degree of tension, and there is even the risk of dehiscence in more fragile areas, or enlargement and hypertrophic scarring, which diverge from optimal healing^4^. 

Careful management with analgesics and even opioids is required, which can slow recovery and consequently delay the return to normal activities. In addition, the need for surgical drains to prevent the accumulation of fluids and collections in the postoperative period can also lead to some discomfort, prolonged recovery time, and impaired mobility, limiting the overall postoperative experience and postural positioning. 

Despite advances in conventional surgical techniques and attentive postoperative care, a proportion of patients still do not express total satisfaction with treatments’ aesthetic results. Some women report that the resulting final appearance is marked by a large scar, limiting them to certain garments, and aesthetically leading to an appearance that may seem artificial and reminiscent of surgery. In addition, there is a growing specific population of patients seeking surgical treatment, comprising women who have minimal or no signs of skin sagging or significant adiposity, but are diagnosed with diastasis abdominis due to factors such as pregnancy. This audience generally does not qualify for traditional abdominoplasty due to the absence of excess skin and usually seeks solutions that do not involve extensive surgical incisions or the postoperative risks and limitations associated with the conventional procedure. Instead, they prioritize treatment that aims for a natural appearance and crave minimal surgical interventions that can effectively treat diastasis abdominis without the stigmas of traditional procedures.

Recently, the advent of robotic surgery has fundamentally changed the scenario of abdominal wall surgeries^6^ ^,^ ^7^. The robotic surgical platform allowed a new level of precision and movement control, overcoming many of the limitations faced in laparoscopic techniques^8^.

This article aims to present a robotic surgical technique specifically for the correction of diastasis abdominis. Using the enhanced capabilities of the robotic platform combined with experience in the treatment of abdominal wall defects, the described technique allows a posterior approach with minimal and minutely hidden scars in the lower abdomen, improving aesthetic results and patient recovery. In addition, this technique not only improves body contour but also prevents larger incisions, skin flaps, and greater wound morbidity, allowing for a paradigm shift in the treatment of diastasis abdominis. We described a step-by-step standardization of this technique as a technical note below.

## METHODS

This article reports a standardized technical note and its step-by-step guide for surgeons who specialize in abdominal wall and robotic surgery performing minimally invasive repair of diastasis abdominis. 

## TECHNICAL NOTE

The procedure involves the use of the 4th generation Da Vinci robotic platform (Da Vinci Surgical System; Intuitive Surgical, Inc., Sunnyvale, CA). The preoperative evaluation of patients undergoing treatment is a critical step to ensure optimal surgical outcomes. A comprehensive evaluation begins with a detailed review of the patient’s medical history, focusing on specific complaints related to abdominal contouring and functionality. It is essential to ask about the complaint in the face of diastasis recti, the size of the separation, the presence of hernias, the thickness of the subcutaneous fat, and any previous abdominal surgeries that may have left significant scars. In addition to clinical evaluation, imaging studies such as CT scans can be utilized to accurately assess the extent of diastasis and any underlying anatomical considerations that may affect the procedure. Good physical examination and imaging are vital in mapping body contour prior to intervention and can aid in preoperative planning and patient counseling of potential outcomes and realistic expectations. In addition, assessing the patient’s body contour and existing scars allows insights into the strategic positioning of incisions and the benefit of combining skin retraction techniques and collagen stimulation, further optimizing aesthetic results.

### Step 1: Patient Positioning

The patient’s position is similar to those commonly performed in robotic abdominal wall surgery, previously described by our group9, in a supine position, arms close to the trunk and allowing flexion of the table to obtain a greater space for hip trocars and minimize the risk of external collision of the robotic arms. In addition, we recommend the insertion of an indwelling urinary catheter to ensure an empty bladder during the operation.

### Step 2: Abdominal cavity access, portal placement, and docking

**Figure 1.**
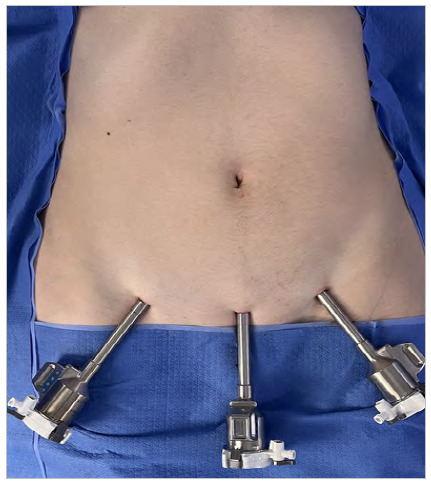

The surgery setup should include three 8mm robotic portals, a 30° robotic endoscope, and robotic instruments, more precisely a monopolar pair of scissors, a fenestrated bipolar forceps, and a needle holder. Pneumoperitoneum is obtained by a Veress needle puncture, and the portals are inserted. The optical robotic portal is placed in a suprapubic position in the midline, and both the left and right portals are placed lateral to the inferior epigastric vessels (Figure 1). The robot is then docked, and the instruments are introduced under vision.

### Step 3: Dissection

**Figure 2.**
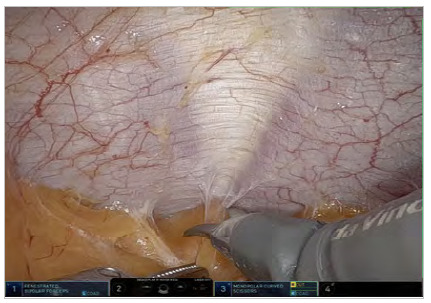

**Figure 3.**
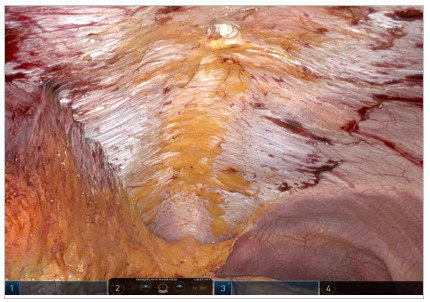

**Figure 4.**
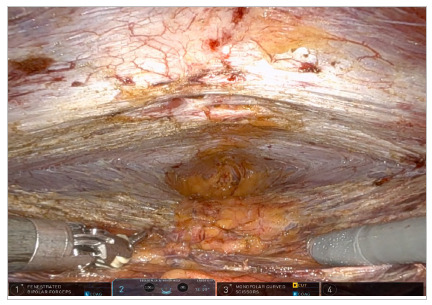

From a caudocranial perspective, an incision in the peritoneum is made very close to the camera in the suprapubic region, creating a peritoneal flap in the pre-transversalis or pre-peritoneal plane initially. Dissection continues cranially and laterally under the impression of the edges of the rectus abdominis muscle, exposing diastasis and possible midline hernia defects. One of the lateral edges of the flap is sectioned to ensure better reach of the instrument in the epigastric region of the abdomen without traction of the structure, and the diastasis and its concomitant hernias are completely exposed in the pre-transversalis plane up to the subxiphoid space (Figures 2, 3 and 4). In cases where concomitant hernias are being treated, to allow for a larger mesh and overlap, we recommend to not section one of the lateral edges of the flap, recruiting the transversalis fascia bilaterally maintaining flap integrity. It is important for the surgeon to be precise and avoid peritoneal punctures during this stage of the procedure, given the particularities of the fatty trident anatomy already described^10^.

### Step 4: Plication, defect correction, and fascial coverage

**Figure 5.**
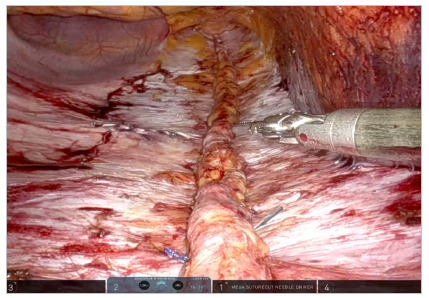

Once the diastasis is completely exposed and possible midline defects have their content reduced, we proceed to the closing and plication part of the diastasis. The image inversion artifice can be performed at any time, depending on the surgeon’s needs, by means of a previously published sequence of maneuvers on the robotic instruments of the patient’s console and cart^11^. Plication is performed using a size 1 nonabsorbable barbed suture in a continuous inverted suture, ensuring intra-abdominal accommodation of the diastasis tissue (Figure 5).

**Figure 6.**
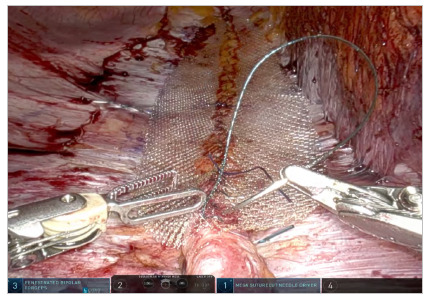

**Figure 7.**
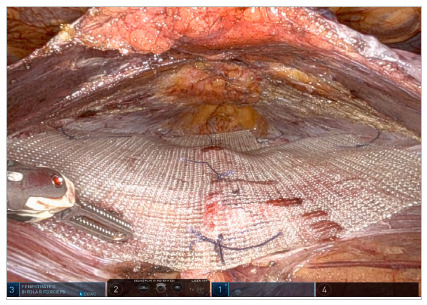

**Figure 8.**
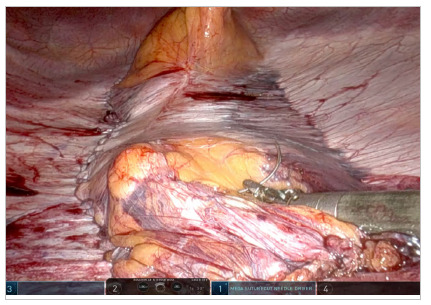

Complete plication and closure of hernia defects are achieved in a craniocaudal direction. In experienced hands, a wide side-to-side dissection of the peritoneal flap can be obtained if the placement of an uncoated mesh covering the entire plication is considered. The space is measured with a sterile ruler and the mesh is allocated and fixed with a suture (Figures 6 and 7). Finally, complete fascial coverage of the dissected space and the plication area is achieved by repositioning the previously dissected flap through a suture (Figure 8).

[Fig f9]

**Figure 9. f9:**
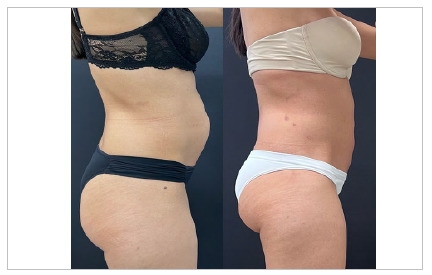

## DISCUSSION

Patients suffering from diastasis abdominis commonly present with a variety of symptoms that reflect both functional impairments and aesthetic concerns. Aesthetically, diastasis rectus abdominis can lead to significant changes in body contour, often resulting in a more flaccid appearance, which commonly distorts body image. Among the most common complaints, they often express dissatisfaction with their abdominal profile, stating that it is projected and uncomfortable, particularly in more waisted clothing or an upright posture.

Conventional abdominoplasty, although effective in removing excess skin and treating abdominal contour, has some limitations that may impact patient satisfaction and recovery^4^. A commonly reported disadvantage is the larger incisions required that lead to indiscreet scars. In addition, patients may face postoperative limitations such as seroma formation, long healing times, and even wound enlargement, as well as prolonged recovery times. In addition, the creation of a new navel can be a challenge, since many patients deem it something artificial, contributing to possible dissatisfaction with the final aesthetic result^12^.

The desire for aesthetically pleasing results without the major stigmas associated with traditional surgeries has fueled interest in alternative approaches, particularly those that allow for functional correction with minimal alteration of the body’s natural anatomy. The robotic technique described represents a significant advance in meeting this scenario, offering not only satisfactory aesthetic results, but also a superior functional recovery compared with conventional methods.

The technique described aims to obtain a repair of the linea alba in a less invasive way, restoring the integrity of the abdominal wall and achieving results that closely reflect the natural anatomy of the pre-gestational patient. Through the robotic platform, precise dissection and sutures are feasible, which are crucial to achieve optimal plication, minimizing the risk of recurrence^10^.

It is important to emphasize that the described technique was studied to be performed in the pre-transversalis plane, achieving a more physiological repair compared with the preaponeurotic, retromuscular, or even purely intraperitoneal ones. Avoiding the pre-aponeurotic plane minimizes further subcutaneous dissection and associated risks such as seroma formation and collections^13^ ^,^ ^14^. In addition, it is less traumatic than retromuscular approaches because it does not violate the posterior sheath of the rectum, thus maintaining the structural integrity of the abdominal wall^15^. This preservation is crucial to prevent abdominal protrusion mechanisms that can arise from the disconnection of the posterior sheath. Finally, in purely intraperitoneal techniques such as Intraperitoneal Onlay Mesh (IPOM), one of the most significant concerns is the formation of adhesions, which can develop between the mesh or by the exposed sutures themselves and adjacent tissues^16^. Consequently, the pre-transversalis approach described above promotes more physiological and anatomical restoration, optimizing results and minimizing disruption of the abdominal wall natural biomechanics (Figure 8).

Another key point of the robotic diastasis abdominal surgery is that it not only achieves functional results but also opens paths for the incorporation of complementary aesthetic procedures. These synergistic approaches can improve the overall outcome by providing patients with a comprehensive solution tailored to their specific needs and aspirations. The ability to combine surgery with other procedures, such as liposuction, allows for the targeted removal of excess fat tissue, leading to better body contouring while also addressing the structural integrity of the abdominal wall. Liposuction, when performed simultaneously with minimally invasive diastasis repair, increases the aesthetic effects of abdominal definition, allowing for more sculpted contours. In addition, other advanced technologies can also be used to reduce skin sagging and improve results, such as Renuvion, Argoplasma, BodyTite, and Morpheus, each with its own particularity in search of tissue improvement^17^. The selection of such specific modalities should be considered with professionals specialized in plastic surgery to ensure their effectiveness and care.

Treatment success is multifactorial, and based on the association of key factors, including a complete preoperative evaluation, with a clear understanding of treatment’s objectives and limitations and the surgeon’s experience with robotic surgery and expertise in abdominal wall surgeries.

## CONCLUSION

This study remains consistent with the current literature and describes a robotic surgery technique for the treatment of diastasis abdominis. Through a technical note, we report the step-by-step for an anatomical landmark-guided approach to the posterior abdominal wall. Importantly, this technique is best suited for patients with low sagging skin who are within an adequate weight range and have aligned their expectations regarding surgical outcomes. Patients who have moderate to significant sagging skin and require substantial skin removal are unlikely to achieve the desired results through robotic repair alone. Consequently, a preoperative evaluation with clear discussion of the patient’s goals and the surgeon’s experience are essential to ensure the most satisfactory results.
